# Developing airport management practices towards net zero emissions: Experiences from the Australian aviation industry

**DOI:** 10.1016/j.heliyon.2024.e41201

**Published:** 2024-12-16

**Authors:** Manori Dissanayaka, Tim Ryley, Bojana Spasojevic, Savindi Caldera

**Affiliations:** a Griffith Aviation, School of Engineering & Built Environment, Griffith University, Brisbane, 4111, Australia; b Cities Research Institute, Griffith University, Brisbane, 4111, Australia; c Griffith Institute for Tourism (GIFT), Griffith University, Brisbane, 4111, Australia; d School of Science, Technology and Engineering, University of the Sunshine Coast, Petrie, 4556, Australia

**Keywords:** Airside ground operations, Airport collaborative decision-making, Carbon accreditation, Emission inventory

## Abstract

Emissions from airport sources degrade air quality impacting community health. While some airports assess air pollution, others assess broader environmental effects, including CO_2_ emissions and noise. Utilising a transition management approach, this paper examines Australian airport practices and develops key sustainable strategies to reduce environmental impacts. After reviewing environmental policies and reports from eight major airports and conducting in-depth interviews with 18 sustainable aviation experts, five key strategies are proposed: 1) Collaborating data-sharing among stakeholders, including airport operators, ground handlers, airlines and air traffic controllers; 2) Evaluating emissions from aircraft ground idling delays; 3) Advancing in the Airport Carbon Accreditation program; 4) Assessing air pollutant emissions directly emitted from airport sources; 5) Maintaining an air pollutant emissions inventory. Airports should integrate these strategies into their environmental policies to support their long-term sustainable goal of reaching net zero emissions by 2050.

## Highlights

• Applying Loorbach's spheres tool in airport management to propose sustainable strategies for mitigating air quality impacts.
• Recommended key strategies: maintaining an air pollutant inventory, data collaboration, advancing the Airport Carbon Accreditation, and assessing ground idling emissions.
• Emphasising the integration of these strategies into airport policies to reduce air pollution and achieving net zero emissions by 2050.
• Highlighting the initiation of emission reduction strategies over refining inventory precision to drive further reductions.

## Graphical abstract

**Figure:**
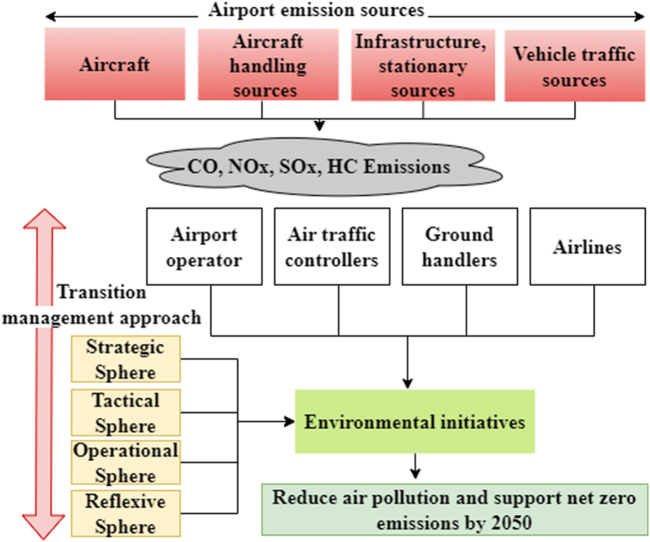

## 1 Introduction

Air pollution is among the most significant threats to human health, according to the World Health Organization (WHO) [1]. Nitrogen dioxide (NO_2_), Ozone (O_3_), Carbon monoxide (CO), Sulphur dioxides (SO_2_), and particulate matter (PM) are the primary pollutants that affect air quality [2]. Exposure to a higher concentration of these pollutants can cause diseases such as stroke, lung cancer, and respiratory problems [3]. WHO recommends comparing current Air Quality Guidelines (AQG) to those set in 2005. In 2019, over 90 % of the global population lived in areas where pollutant concentrations exceeded the 2005 AQG [1]. Airport operations contribute to degrading the air quality. Nevertheless, there remains a notable deficit in public awareness and support for initiatives to mitigate air pollution [4].

The International Civil Aviation Organization (ICAO), the UN's specialized agency for the aviation industry, has formulated sustainable aviation policies. At the 41st ICAO Assembly in 2022 [5], member states committed to achieving net-zero carbon emissions by 2050 for international aviation. Additionally, ICAO urged member states to conduct research and development to mitigate air quality and GHG impacts. The aviation industry's long-term sustainable goal omits domestic and general aviation, focusing primarily on international flights. This paper proposes strategies for improving air quality, which support the achievement of the long-term goal of net-zero emissions by 2050.

### 1.1 Airport sources influencing air pollution

**Fig. 1:**
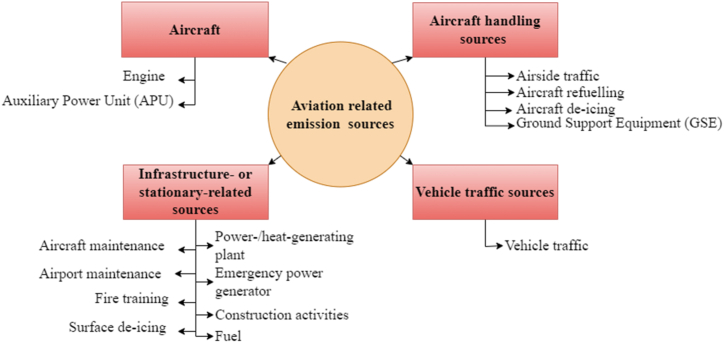
Airport emission sources.

Source: Adapted from the International Civil Aviation Organization airport air quality manual [6].

Beyond aircraft main engines, the airport emission sources depicted in Fig. 1 impact air quality. These airport emission sources are owned and operated by stakeholders, including airport operators, airlines, ground handlers, and air traffic controllers. Since the aircraft's main engine contributions towards emissions are much greater, emissions from other sources have received relatively less consideration [7]. Mazaheri, Johnson and Morawska [8] evaluated NOx and PM emissions from Brisbane Airport, Australia, focusing solely on larger aircraft. However, airports should assess all emission sources to address air pollution comprehensively.

Few researchers considered emissions from other airport sources. Winther, Kousgaard [9], Henry-Lheureux, Seers [10], Xu, Fu [11] adopted ICAO-recommended APU durations (45 min for short-haul flights and 75 min for long-haul flights [6]) instead of actual operational values. Henry-Lheureux, Seers [10] analysed GSE emissions at Montreal's Pierre Elliott Trudeau International Airport (YUL), using Zurich Airport data per ICAO recommendations [6] due to YUL-specific data unavailability. Researchers used ICAO-suggested values to estimate emissions from airport sources with insufficient operational data, potentially resulting in over- or underestimation of actual emissions.

### 1.2 How ground idling delays contribute to air pollution

Aircraft ground idling delays consume additional fuel, causing more emissions. Many researchers have evaluated the economic costs associated with aircraft delays [12,13], but environmental impacts from delays have been less researched. A minute of aircraft ground idling delay is estimated to involve between 1 kg and 4 kg of fuel consumption [14]. Miller, Minogue and Clark [15] found that the taxiing phase (ground idling) duration in US domestic aviation is growing faster than the airborne phase duration. This is projected to escalate with future demand. Hence, emissions from ground idling delays have a substantial impact on local air quality.

### 1.3 How airports assess air pollution

The aviation industry has a globally accepted standard method, Airport Carbon Accreditation (ACA), to assess airports' climate impact [16]. While airports can voluntarily adopt ACA to assess Greenhouse Gas (GHG) emissions, it does not address air pollutant emissions impacting air quality. Some researchers applied the Intergovernmental Panel on Climate Change (IPCC)'s methods to assess air pollutant emissions [[17], [18], [19]]. Synylo and Duchene [20], and Zhu, Hu [21] identified deficiencies in these methods, proposing improvements to the emission factors and fuel flow. At Melbourne Airport, air quality monitoring stations continuously measure atmospheric pollutant concentrations. However, these measurements are often influenced by external factors beyond the airport's control and boundaries [22]. At Sydney Airport, air pollution from aircraft, GSE, and APU were assessed for the year 2016 using the United States Federal Aviation Administration's (FAA) Aviation Environmental Design Tool (AEDT) model [23]. A globally accepted program for assessing air pollutant emissions is still lacking, necessitating further theoretical and empirical research on airport air quality. Given the direct impact on nearby communities, examining airport influence on air quality is crucial. This paper aims to evaluate the practices of Australian airports regarding air quality and proposes environmental strategies to enhance airport policies.

## 2 Materials and methods

An exploratory approach was used to evaluate Australian airport practices and delve into their environmental strategies [24]. Initially, major airports in Australia were selected. Annual reports and environmental policy documents from these airports were reviewed for background information. This was followed by interviews with selected airport personnel for deeper insights.

### 2.1 Airport data collection from documents and interviews

**Fig. 2:**
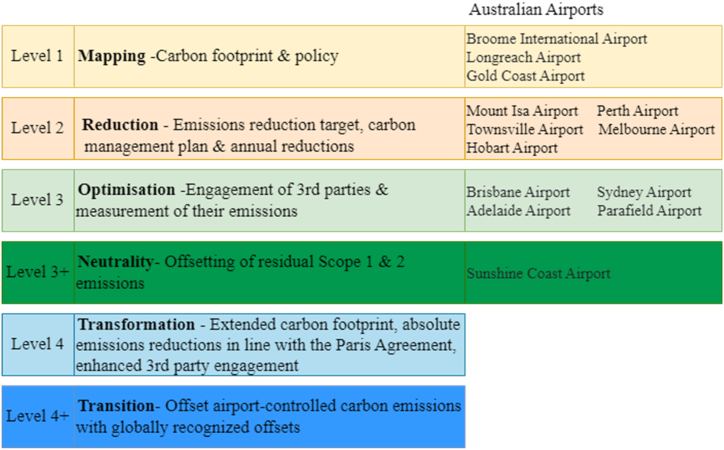
The six levels of Airport Carbon Accreditation and the standing of Australian airports.

Source: Adapted from Airport Carbon Accreditation Annual Report 2021–2022 [26].

Data collection was conducted with a focus on eight major international airports in Australia. Table 1 shows selected airports with passenger traffic as a percentage of total international passenger traffic in the 2018–2019 financial year before the COVID-19 pandemic impacted them. Australia has more than 170 airports [25], although many of them are small and in remote regional areas. According to Table 1, eight selected major Australian airports account for 84 % of air passengers while others account for the remaining 16 %. Therefore, the chosen eight airports are a good representation of all the Australian airports according to the passenger volume. Another reason for choosing these airports is their participation in the Airport Carbon Accreditation (ACA) program and their existing maintenance of a globally verified CO_2_ emission inventory. ACA program comprises 6 certification levels (Fig. 2). As per the May 2021–May 2022 annual report, 395 airports adhere to the program, with 16 already at the 'Transition 4+' level [26]. Among them, 13 Australian airports participate in, their positions within the 6 levels shown in Fig. 2. Table 2 shows the list of published documents reviewed as secondary data from the selected airports.

**Table 1:** International Passenger Traffic through Australian International Airports, 2018–2019 (in order of size).

Airport	Level in Airport Carbon Accreditation Program	Total Passenger Movements	% of Total Passenger Movements
1. Sydney Airport	3	44375769	27
2. Melbourne Airport	2	37057190	23
3. Brisbane Airport	3	23623245	14
4. Perth Airport	2	12405381	8
5. Adelaide Airport	3	8368177	5
6. Gold Coast Airport	1	6413815	4
7. Hobart Airport	2	2725559	2
8. Sunshine Coast Airport	3+	1257561	1
Other Airports		26870971	16
Total		163097668	100

**Table 2:** Aviation policy documents reviewed in the paper.

Organisation	Published documents
Sunshine Coast Airport	Sunshine Coast Airport – Environmental Policy 2022, Carbon Management Policy 2022
Adelaide Airport	Environment Policy- 2019, Sustainability Policy- 2018
Brisbane Airport	Brisbane Airport Corporation Annual Report 2022
Sydney Airport	Sydney Airport Environment Strategy 2019–2024
Gold Coast Airport	Queensland Airports Annual Report 2022, Gold Coast Airport master plan 2017
Hobart Airport	Energy Use Reduction Policy −2022
Perth Airport	Perth Airport Master Plan 2020
Melbourne Airport	Melbourne Airport Environment Strategy 2018
Airservices	AirServices annual report 2019–2020, Environmental sustainable strategy 2020–2026

Interviews were chosen as a suitable method for gathering empirical data and acquiring valuable insights into the research phenomena [29]. The interview procedure outlined adheres to the principles stated in the Declaration of Helsinki, ensuring the ethical treatment of human participants [30]. Before their involvement in the study, participants provided informed consent. There were 18 interviews conducted online (Microsoft Teams) between January and May 2023. The interviewees were experts in sustainable aviation. Interviewees were searched on LinkedIn and airport websites. Snowball sampling [31] was used to identify further potential interviewees suggested by initial participants. Interviews continued until data saturation was achieved [32].

Table 3 shows a summary of the interviewees. As most participants preferred not to have their responses attributed to their organisations, no company or personal names were provided. Interviewee information is de-identified. All interviews were video recorded and automatically transcribed. The research team then reviewed the transcriptions. Griffith University's ethical procedures (GU ref no: 2022/841) were followed for these interviews. The interview questions were designed to investigate two main areas: (1) the airside ground operations at airports, and (2) the environmental strategies and practices implemented to manage emissions and air quality. Open-ended questions (Appendix 1) were included to have detailed responses and rich qualitative data. Appendix 1 presents a structured set of predetermined questions that guide the conversation while ensuring consistency across interviews. Dissanayaka et al [33] conducted a systematic literature review on methods for evaluating aircraft emission impacts on air quality, which guided the development of the preliminary interview questions for this study. Cooper and Schindler [34] and Kvale [35] guided the structuring and conducting of effective interviews, ensuring the relevance and methodological rigor of the questions. Follow-up questions were employed to delve deeper into specific areas to clarify and expand upon responses provided by the interviewees. This methodological design ensured that while core areas of interest were systematically addressed, unique and context-specific insights were also captured, thereby enriching the overall data collected.

**Table 3:** List of interviewees.

Participant ID	Role Description	Interview Pseudonym
1	Manger- Aviation support	Airport A, P1
2	Head of airside operation	Airport A, P2
3	Group member, Net zero, renewable energy	Airport B, P3
4	Sustainability Manager	Airport C, P4
5	Senior Leader in Environment and Sustainability	Airport D, P5
6	Environment Manager	Airport D, P6
7	Environmental Manager	Airport E, P7
8	Executive General Manager People, Culture and Environment	Airport F, P8
9	Sustainability Analyst	Airport G, P9
10	Service Manager	Airport G, P10
11	Senior Manager - strategy and operations	Airport G, P11
12	Head of Environment and Sustainability	Airport H, P12
13	Air Traffic and Meteorology Manager	Airline A, P13
14	Manager, flight dispatch	Airline A, P14
15	Airline Operations Manager	Airline A, P15
16	Trust Ambassador	Airline B, P16
17	Approved ACA verifier	Third-party organisation, P17
18	Air traffic controller	Airservices, P18

### 2.2 Thematic analysis of interview transcripts

**Fig. 3:**
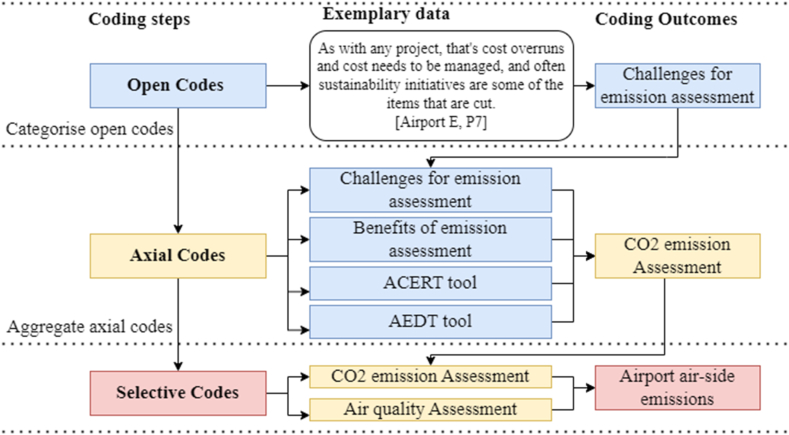
Coding diagram.

Annual reports and policy documents were initially examined from the eight major Australian international airports to identify background information and formulate interview questions. Interview transcripts were thematically examined to identify, analyse, and report themes within data [36,37]. NVivo (Release 1.7.1) software was used to organise, compare, and thematically analyse the data [[38], [39], [40]]. The transcripts were oranigsed in NVivo and then repeatedly reviewed to grasp the content and note initial impressions and standout ideas. The transcripts were segmented into chunks, each conveying a piece of information, and labeled with a corresponding code data [41]. Axial coding techniques were then used to iteratively deduce the 45 open codes into 13 major categories, establish connections between open codes, and develop selective codes [32]. Selective coding focused on narrowing down and aligning the themes with the research question (Fig. 3). Several panel meetings were held with co-authors to select the most compelling and representative excerpts for this study [42]. The first author developed an audit trail, a systematically maintained documentation system that tracks the sequence of activities, decisions, and procedures. This ensures transparency and credibility of exploratory findings [43]. The thematic analysis highlights five key emergent themes that support the identification of airport strategies that relate to air quality impacts.

A sample size of 18 interview transcripts was deemed sufficient for NVivo analysis, enabling the identification of five themes. This adequacy was due to the depth of the interviews, the homogeneity of the participant group, and the complexity of the research question, with data saturation reached at 18 interviews. Crouch and McKenzie [44] argued that a sample size of fewer than 20 is often sufficient for qualitative research aimed at deep understanding rather than broad generalization. However, due to the adherence to ICAO standards in the aviation industry, 18 transcripts were sufficient to achieve data saturation.

## 3 Theory

Transition management [45] as a governance approach is commonly used in sustainability studies within organisations, particularly in energy [46], water [47], and tourism [48]. It emphasises the process of moving from unsustainable to sustainable practices with stakeholder support. However, its application has been limited within the domain of airport management. Furthermore, in many states, national authorities, often with regional and local authorities, establish their own air quality principles for maintaining acceptable air quality conditions [6], which airports voluntarily follow. However, airports lack clear standard environmental strategies similar to these for airport operations. By employing this approach in airport management, the paper aims to provide airport stakeholders with guidance to improve air quality and support the achievement of long-term sustainability goals, thereby strengthening their environmental policies.

Loorbach [46] introduced the following four spheres as a transition management tool, which was utilised in this paper. This tool facilitates the achievement of long-term, uncertain goals through practical, incremental steps.

a) The strategic sphere involves formulating long-term sustainable goals within the airport, encompassing existing airport regulations and policies regarding air quality.
b) The tactical sphere involves collaborative efforts among airport stakeholders (ground handling companies, air traffic controllers, airlines) to achieve sustainability goals.
c) The operational sphere focuses on short-term actions, considering current sustainable strategies to reduce air pollution.
d) The reflexive sphere evaluates ongoing strategies, including discussions on barriers to current sustainable strategies.

## 4 Results and discussions

**Fig. 4:**
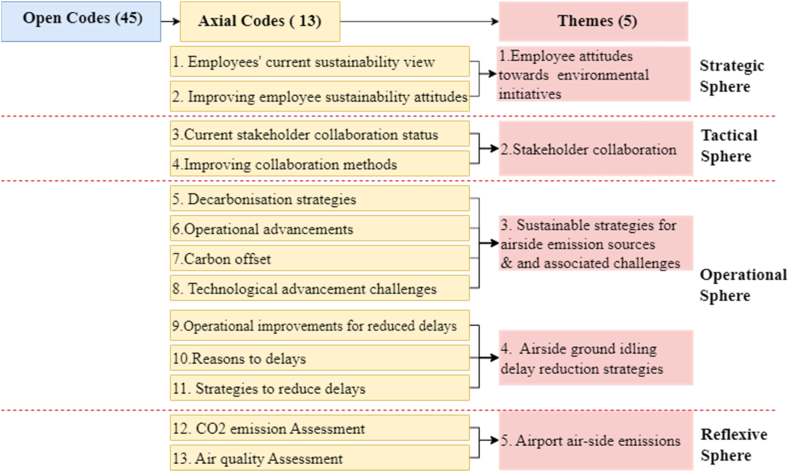
Main categories of primary codes and associated key themes in the analysis.

As shown in Fig. 4, the five themes were categorised according to the framework of Loorbach's (2010) four spheres, providing a structure to the results section (Sections 4.1 to 4.4).

### 4.1 Strategic sphere: airport policies related to air quality

Airports develop and maintain their own environmental strategy documents to work towards the long-term sustainable goal of achieving net zero emissions by 2050, including air quality initiatives. These documents outline action plans guiding each employee. According to the reviewed airport documents, the following steps were taken by Australian airports to improve air quality. As per Gold Coast Airport's 2017 Master Plan, they compared air quality results from the nearest air quality monitoring station in 2014 against the National Environment Protection Measure (NEPM) and Airport Environment Protection Regulations 1997 (AEPR) requirements. According to these results, the regional air quality met the specified guidelines [49]. In 2016–17, Melbourne Airport undertook an air quality impact assessment and the results did not represent a significant air quality issue compared with national and Victorian air quality standards [22]. Even though, the air quality measurements currently fall below the established standards, ongoing assessments and regular comparisons remain essential, given the rapid growth in aviation demand.

#### 4.1.1 Theme 1: Employee attitudes towards environmental initiatives

Airports require a dedicated team to work together to implement environmental initiatives. Airport D P6 mentioned that they maintain an airport environment strategy document to inform the organisation and ensure everyone understands their role. Maintaining an emission inventory is a positive indication that the organisation values and prioritises environmental responsibility. During the interview with third-party organisation P17, an ACA-approved verifier with extensive experience across multiple airports, it was noted that the highest-level airports within the ACA program have an exceptionally committed team. Some airports believe that the responsibility of maintaining the emission inventory falls to a few individuals within the organisation. According to the following response, it is evident that this emission estimation process should be a collaborative effort of all the staff in the organisation.

“One of New Zealand airports has the advantage of having an incredibly dedicated and motivated sustainability team. So everyone knows what they need to contribute to the reporting. Sustainability is part of their culture, whereas, with other airports, you will see that the reporting is largely left to one person, maybe two people, and it can be more difficult to get that information. Not impossible, but there are still other functions within the airport.” [Third-party organisation P17]

The following response shows the organisation's commitment and emphasis on environmental initiatives. It highlights a common practical challenge that airports face: environmental initiatives are often the first to be cut when cost-saving measures are needed. This indicates a potential conflict between financial management and environmental objectives. However, organisations must first adjust their attitudes towards environmental responsibility within the organisation. Subsequently, the organisation can motivate and engage other airport stakeholders in adopting environmental initiatives.

“As with any project, that's cost overruns and cost needs to be managed. And often sustainability initiatives are some of the items that are cut when the cost-saving measures need to be implemented. So, it's just around making sure that's appropriately acknowledged and accounted for in the initial.” [Airport E P7]

### 4.2 Tactical sphere: how airport stakeholders work towards air quality

Educating both staff and tenants is key to getting their support for achieving the environmental initiatives. As per the 2022 annual report of Brisbane Airport Corporation, 99 % of their overall emissions fall within Scope 3, which is owned by airport stakeholders [50]. The primary contributors to Scope 3 emissions consist of aviation fuel, ground servicing equipment, passenger transportation to the airport and third-party electricity consumption. Therefore, airport stakeholders should proactively endorse environmental initiatives, given their significant role in emissions. Gold Coast Airport encourages its tenants to decrease energy usage through leasing agreements and audits [51]. According to the airport environmental strategy reports, Sydney Airport [23] and Melbourne Airport [22] continue discussions with Airservices Australia and other key stakeholders on minimising aircraft taxiing times, idling times, and engine usage. These actions demonstrate that certain airports are already taking steps to enhance collaboration among stakeholders to reduce air pollution.

#### 4.2.1 Theme 2: Collaboration among stakeholders

The air traffic controllers, ground handling companies, and airlines are key stakeholders supporting airport airside ground operations, each with its own environmental targets. However, airports face limitations in influencing or pressuring these stakeholders. Some stakeholders may need to undertake additional tasks beyond their usual responsibilities when providing relevant data for emission assessments, potentially contributing to their limited level of support. Nevertheless, collaboration among all stakeholders is essential, as environmental responsibility is a shared obligation among them.

*"*I would say is the willingness of third parties. We can see their pathway and their targets. But they will only do what they want to do. We can only go so far as to educate and collaborate, but they will always do what they wanna do. That's a barrier." [Airport C P4]

“We're just waiting on Level 3 accreditation approval, but that requires us to do is not only reduce the emissions which we have control over but also that we influence those business partners and the Scope 3 emissions. So, obviously we can't change or make decisions around airline decisions cause obviously that has the greatest amount of emissions we can work with them as best we can to consider how we can encourage them to think about their emissions” [Airport F, P8]

Airports A and D have proposed the implementation of Airport Collaborative Decision Making (A-CDM) to facilitate data collaboration among aviation stakeholders. A-CDM aims to enhance airport efficiency by optimising resources and increasing the predictability of air traffic. Specifically, it concentrates on improving aircraft turn-round and pre-departure processes to facilitate better decision-making, thereby reducing ground idling delays and enhancing overall efficiency. By improving airfield efficiency, airports can concurrently decrease fuel consumption and emissions. The improved predictability allows for smoother traffic flow, which reduces congestion both in the air and on the ground, further lowering emissions. However, airport stakeholders should work more transparently and collaboratively with the A-CDM Network Manager, exchanging accurate and timely information.

" For delays A-CDM is probably one of the bigger strategies that we are implementing, and because that's network vice that's a whole of Australia, which is implementing that should have a large effect. That will reduce a significant amount of fuel burn for aircraft holding at the runway intersection so that system will work by the aircraft will hold at the gate rather than push back or taxiing with engines on." [ Airport A, P2]

**Strategy 1) Collaborating data-sharing among stakeholders, including airport operators, ground handlers, airlines, and air traffic controllers**

Some airports have adopted A-CDM to enhance the efficiency and quality of operations [52]. Zheng, Miao [53] outline the limitations of A-CDM implementation, including insufficient capability for automatic data acquisition, challenges in information sharing and exchange, redundancy in system processes, and a lack of advanced big data analysis and mining capabilities. Despite these challenges, implementing A-CDM remains an effective sustainable strategy that can be integrated into the environmental policies of airports and other stakeholders.

### 4.3 Operational sphere: short-term actions, considering current sustainable strategies

In line with Loorbach's (2010) operational sphere, attaining long-term sustainable goal necessitates a collaborative effort with short-term targets that engage all stakeholders. In this section, short-term activities undertaken by airports and stakeholders to mitigate the impact on air quality were examined. Two themes emerged from the interviews: sustainable strategies for airside emission sources and strategies for reducing airside ground idling delays.

#### 4.3.1 Theme 3: Sustainable strategies for airside emission sources and associated challenges

Strategies related to Auxiliary 10.13039/100028466Power Units (APUs), Ground 10.13039/100028466Power Units (GPUs), Ground Support Equipment (GSE), and airside vehicles are considered under this theme. Electrifying GPUs and GSE is a popular strategy that eight airports expect to implement. Currently, these airports use diesel-powered GPUs and GSE that are owned by ground handling companies. By incorporating electrified GPUs, the operating time of APUs can be shortened, resulting in fuel savings. However, the airport operators should provide infrastructure when electrifying. According to the following response from Airport D, it is evident that some airports continue to hesitate in electrifying GPUs and GSE, thinking about the cost of recovery. Airport F mentioned that it is challenging for them, being a smaller airport, to bear the enormous capital cost of implementing new technologies. However, despite the higher initial cost of electrifying GPUs, given its sustainable benefits, airlines, ground handling companies and airports should collaboratively accept and share the cost.

" It is our responsibility to install the infrastructure needed, the Ground Power Units and the preconditioned air. However, we should have a charging mechanism to recover the cost … if the pricing is too expensive in comparison to the cost of jet fuel and the airline would probably just continue to utilise their APUs because it's cheaper." [Airport D, P5]

Using alternative fueled vehicle transport within the airside, installing LED lighting systems, using solar power, and carbon offsetting are the other sustainable strategies highlighted in the interviews. Shang and Lv [54] and Shang, Ling [55] highlight the importance of reducing energy intensity and transitioning to low-carbon energy sources, which can inform policies and practices aimed at improving air quality in and around airports, thereby contributing to more sustainable and healthier urban environments. However, implementing these strategies may encounter challenges such as logistical adjustments, initial investment costs, technological constraints, and regulatory complexities. While these sustainable strategies offer promising solutions to reduce environmental impact, addressing these challenges effectively is crucial for successful implementation.

#### 4.3.2 Theme 4: Airside ground idling delay reduction strategies

Currently, prioritising delays and fuel efficiency is a critical concern for airlines. Ground idling delays may stem from several reasons, including weather conditions, emergencies at the aerodrome, changes in wind and other traffic. Aircraft ground idling delays, with the engine running, can occur either at the gate or during the taxiing phase. Airline B P16 and Airservices P18 mentioned that an incident where passengers seated do not match with the passengers checked-in is a common delay reason at the gate that the airline is responsible for. According to the following response, it is evident that this delay is resulting in a significant waste of time.

“The flight requires certain paperwork on board before departure. This is for several reasons, the main reason is that in the event of an incident, there has to be current paperwork showing the number of people on board and where they are seated. This is for emergency services to account for people in the event of a crash. So, if someone fails to board, this can create delays when it comes to updating and reprinting the paperwork. On that note, if someone doesn't board, the crew have to remove any checked-in baggage that could be stored in the aircraft.” [ Airservices P18]

Checked-in baggage is loaded onto the aircraft according to a baggage loading plan to ensure proper weight distribution. Removing checked-in baggage due to an incident requires operating APUs or GPUs at the gate, resulting in additional emissions as passengers are already onboard the flight. Airlines should focus their attention on reducing this common delay, as it benefits passengers and contributes to the airline's overall efficiency and reputation. Furthermore, airlines should assess the environmental cost of these delays as an environmental responsibility.

The ground idling delays can be reduced by proper communication of information among relevant parties including airlines, air traffic controllers, ground handlers and weather services. Airport operators can also contribute to reducing ground idling delays by providing proper infrastructure. Airports A, C and E mentioned improving taxiway designs to reduce aircraft taxiing time on the ground, thereby directly mitigating ground idling delays.

“We work directly with air traffic Services and the Bureau of MET. so that we can forecast with their plan either not weather coming through or manpower resource shortages from an air traffic services standpoint. So that we can delay the flight. So, you don't depart on time. But you're not operating the aircraft either, so we worked through initiatives such as that. but a lot of data-focused as well, so understanding what the data is saying. So that we can plan responsible turn times over aircraft to give us enough time to manage any disruption like that in the system in the network.” [Airline A, P15]

**Strategy 2) Evaluating emissions generated due to aircraft ground idling delays**

Assessing emissions from aircraft ground delays helps airports and airlines understand their impact on local air quality, enabling targeted interventions to reduce pollution. These assessments provide data to inform policies aimed at reducing delays and emissions, potentially leading to stricter regulations on aircraft ground idling times and the adoption of more efficient ground operations, which can result in significant cost savings for airlines. However, emissions assessments can be complex and costly, requiring advanced modeling tools, data collection, and expertise, which may burden smaller airports. Incomplete or inaccurate data can hinder effective interventions. Additionally, the immediate impact on emissions and air quality may be limited if changes are not implemented promptly, potentially frustrating stakeholders. Emission inventories track emissions from sources but cannot identify emissions caused by delays. Therefore, airlines should take the initiative to calculate the environmental impacts of delays, as this can greatly contribute to improving their operational efficiency.

### 4.4 Reflexive sphere: evaluation of current emission assessment and obstacles

#### 4.4.1 Theme 5: Airport airside emissions

This section analyses how the selected airports measure CO_2_ emissions and air quality. Current emission assessments were examined to identify areas for improvement and to propose potential strategies that other airports could implement in future assessment processes.

##### 4.4.1.1 CO_2_ emission assessment

Airports choose to participate voluntarily in the Airport Carbon Accreditation (ACA) program, acknowledging the importance of its associated benefits.

“We get a couple of opportunities from the ACA program. The first one is an external recognition, but firstly verification of our data. You know, having externally assured data is important to us in the airport, particularly for key types of metrics like carbon. Secondly, it helps us reduce our operating expenses by reducing scope one and two emissions through like Scope two energy efficiency measures on-site solar, things like that.” [Airport D, P5]

These airports receive external recognition and cost savings through improved operational efficiency. As they progress within the ACA program, airports are encouraged to employ various strategies aimed at decreasing their emissions. Therefore, the ACA program presents a commendable approach for every airport to adopt to reduce its environmental footprint.

“So, at level one, airports don't need to show emission reduction. It's just mapping. From levels onward, Level 2, Level 3, and Level 3+, you need to demonstrate a reduction against a 3-year rolling average. If you don't demonstrate that you get downgraded unless you get a special big event like the Olympics. or Commonwealth Games or something like that.” [Third-party organisation, P17]

The ACA focuses on implementing actions to lower emissions rather than putting a strong emphasis on maintaining a highly accurate CO_2_ emission inventory. To attain a reduction compared to a three-year rolling average of emissions, the airport must put into action several new emission reduction strategies. However, these airports do not explicitly quantify the outcomes of their implemented strategies. Therefore, establishing a methodology for quantifying emission reductions resulting from implemented sustainable strategies holds greater importance. The actual environmental benefits achieved through these strategies can serve as motivation for stakeholder engagement in sustainable initiatives.

According to the interview results, one airport uses the FAA's Aviation Environmental Design Tool Aviation Environmental Design Tool(AEDT) for CO_2_ emission assessment, while seven other airports use the Airport Carbon and Emissions Reporting Tool (ACERT).

"For GSE, we don't use fuel data from the ground handling agencies. The AEDT tool simply does a modelling estimate. It is assumed that all aircraft will require the full set of GSE provided in the AEDT tool ….and then for estimating emissions to do with APUs, it uses a standard 13-minute duration for APU runs.” [Airport D, P5]

Records of APU and GSE fuel usage or operational duration are not maintained by these eight airports, as other stakeholders own these sources. Consequently, emissions from these sources are estimated using available assumptions. However, if the ACA program could access airport-specific average operational values for these emission sources, it could enhance the accuracy of the estimates. Hence, airlines and ground handling companies should monitor their use of APUs and GSE, and evaluate emissions to understand their environmental impact.

The ACERT tool can be used easily without emissions or environmental expertise [36]. ACERT uses simple methods and generates an approximate emission value. Therefore, CO_2_ emission inventory can be generated with the assistance of the existing personnel. This is another piece of evidence indicating the ACA program emphasises reducing emissions rather than maintaining an exact emission inventory. Nonetheless, Airport C, E and G have stated their commitment to enhancing data collection for future emission assessment. Eight airports have enlisted the assistance of external consultants for these assessments. These actions demonstrate airports' commitment to more precise emission assessments. Therefore, this indicates that airports should be aware of the ACA's objectives of implementing emission reduction strategies rather than a highly accurate CO_2_ emission inventory.

“I think it's important to note that the approximations are fine. They still serve as a baseline or a feed his source of information for future projects. And I think that it is much more important to have a general idea than spending too much time on getting it 100% accurate. Ultimately, as long as you use the same methodology and the same calculation if you implement a project, the reduction is the same, and I think that's more important. It's more the absolute reduction or the percentage of reduction that you can achieve. So, I think it's much more important to focus on what can we do to bring those emissions down rather than, Oh yeah, we need another half percent accuracy.” [Third-party organisation, P17]

Interviewees from the eight airports mentioned data compilation from multiple stakeholders as the primary barrier to the ACA program. Several manual tasks currently impede emission assessment. Automating these processes would enhance efficiency and effectiveness. However, data can be sourced from stakeholders by raising their awareness of the importance of the current assessment process.

**Strategy 3) Advancing the Airport Carbon Accreditation (ACA) program**

By following this framework, airports are guided in the adoption of cleaner technologies and sustainable practices, which can, in turn, positively affect local air quality. Furthermore, proactive carbon management through ACA can position airports advantageously for future regulatory requirements concerning carbon emissions and air quality standards. However, the initial costs of following the ACA program can be high, including expenses for infrastructure upgrades, new technologies, and audits. Additionally, the improvements in air quality resulting from these efforts may be indirect and may not be immediately evident or quantifiable.

##### 4.4.1.2 Air quality assessment

Several airport activities have the potential to influence air quality. However, air quality assessment is not as widely recognised as CO_2_ assessment. According to the interviewees, four airports conduct regular air quality monitoring, while the other four airports assess air quality only when necessary.

Airport D, P6 and Airport G, P9 stated that they lost their continuous evaluation of air quality due to circumstances that were beyond their control. According to the interviewees, the airport is typically affected by bushfires, controlled burns and road traffic, and they are outside the airport's control and boundary. Currently, these airports obtain air pollutant emissions data from public air quality monitoring stations. Then, these data are compared with Australian national environmental protection measures for ambient air quality. However, airports cannot identify their exact contribution toward air quality by observing these data as they include emissions from sources outside the airport boundary.

Air pollution around airports is influenced by several factors beyond the airside sources (Fig. 1). Meteorological conditions such as wind speed, temperature, and atmospheric stability, are critical in determining pollutant dispersion and concentration [56]. Additionally, urban landscape patterns [57] and the design of airport infrastructure, including access roads and parking, also affect traffic flow and congestion, thereby influencing air pollutant emission levels. Vehicular traffic, both airport-related and general [58], along with traffic congestion around airports, contributes to air pollution. Climate change is expected to worsen air quality around airports in several ways. Due to rising temperatures, increased bushfire frequency and severity will release large amounts of PM2.5 and PM10, which can travel long distances and degrade air quality [59]. Additionally, heatwaves and higher temperatures can enhance the formation of ground-level ozone [60]. Furthermore, alterations in precipitation patterns may impact pollutant removal, as variations in rainfall influence the washout of PM and ozone, in turn affecting air quality [61]. Thus, many factors outside the direct control of airports profoundly influence the surrounding air quality, complicating efforts to precisely quantify the airport's specific contribution to atmospheric pollutant concentrations.

Airports follow different strategies toward air quality, as they need to improve the reliability of the data captured for proper assessment. Airport D, G and H maintain their own air quality monitoring stations within their airport boundary. Airport D installed some air quality sensors around the boundaries to enhance data reliability. Airport G uses the expertise of a consultant when assessing air quality impacts. However, all these strategies should be implemented with the goal of estimating approximate air pollutant emissions. Emphasis should be placed on air pollutant emission reduction strategies rather than prioritising a highly precise air pollutant emission inventory.

“So, we pick up a lot of that citywide pollution with our monitors. That's not necessarily from the airport. So that's one of the measures that we're sort of doing is trying to understand our exact contribution. And also, through a lot of these decarbonisation, you know, initiatives that have a direct correlation to air pollutants. Yeah, by reducing carbon emissions, there's no choice but to also reduce air pollutants.” [Airport D, P6]

Airports A and C expect their influence on air quality to be lower than the standards of Australian national environmental protection measures for ambient air quality. Therefore, they mainly focus on decarbonisation activities, believing that they automatically reduce air pollution. However, given the rapid growth of aviation demand, ongoing assessments and comparisons with established standards are essential to understand long-term air quality trends for future planning and decision-making.

**Strategy 4) Assessing air pollutant emissions directly emitted from airport sources**

Direct assessment offers precise data on emissions from airport operations, enabling targeted mitigation strategies and ensuring compliance with environmental regulations by providing concrete evidence of emission levels. It helps identify major pollutant sources, facilitating focused improvements and setting benchmarks for performance enhancement and progress tracking. However, this approach may also lead to increased regulatory reporting requirements and additional administrative burdens.

**Strategy 5) Maintaining an air pollutant emissions inventory**

Maintaining an emissions inventory enables long-term trend tracking, pattern identification, and evaluation of reduction strategies. It supports benchmarking against industry standards, aids in risk assessment, and enhances transparency with stakeholders by providing clear data. However, challenges include ensuring data accuracy and consistency due to multiple sources and owners, potential non-compliance risks, and the limitation of providing only a snapshot of emissions, which may not capture variations or emerging issues.

**Fig. 5:**
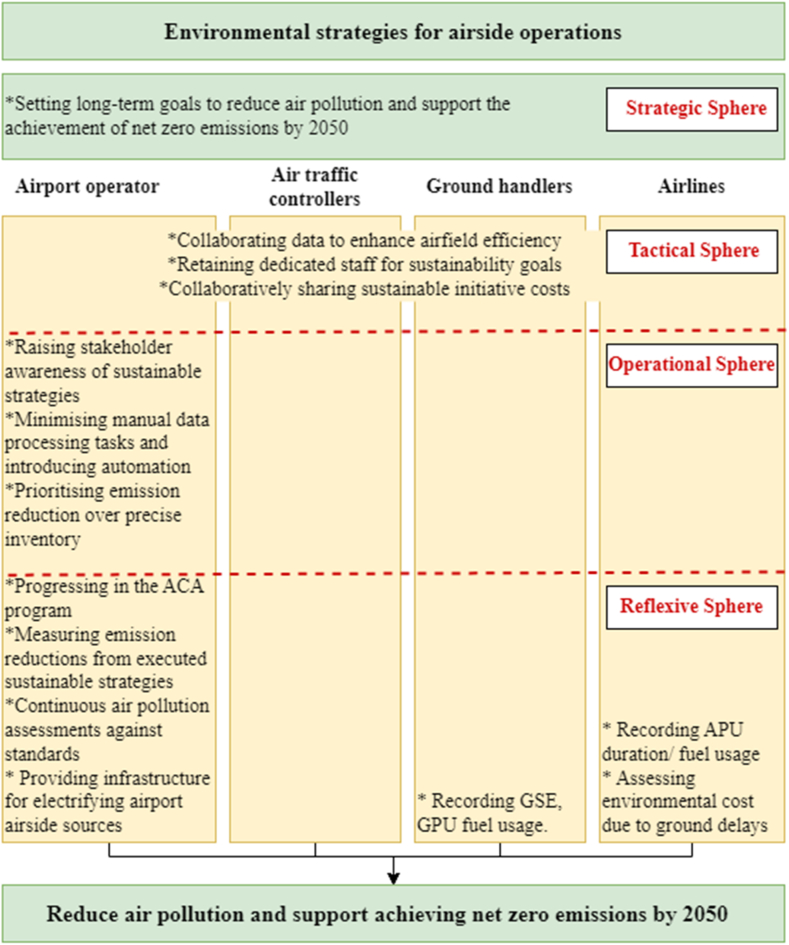
Environmental strategies for airport airside ground operation.

Synthesising key findings from the airport documents and interviews, Fig. 5 summarises potential environmental strategies related to air quality pertinent to achieving net-zero emissions by 2050, focusing exclusively on airport airside ground operations.

In examining strategies adopted by other airports globally to mitigate air pollution, Christodoulakis, Karinou [62] proposed developing specialized electric vehicles to tow aircraft during the taxiing phase at Athens International Airport, which holds a level 4+ certification in the ACA program. A ground-based electric network will power these vehicles. Dong and Chen [63] investigated air pollution around 59 major airports in China and suggested that policymakers address the increasing impact of airport commuting due to aviation demand. Recommended strategies include constructing metro or railway connections between urban areas and airports, and imposing taxes on vehicles commuting to airports. Kansai International Airport, which holds a level 4 certification in the ACA program, follows several environmental initiatives to reduce its impact on air quality. These initiatives include replacing airside vehicles with eco-cars, such as electric, fuel cell, natural gas, hybrid, plug-in hybrid, and ultra-fuel-efficient vehicles [64]. Additionally, ground-handling companies use hydrogen-powered forklifts. To further reduce vehicle idling times, the airport displays signs and posters in parking lots and public areas. These efforts collectively contribute to the airport's commitment to environmental sustainability.

### 4.5 Limitations of the study

This study focused primarily on Australian airports participating in the ACA program. Future research should extend this investigation to airports globally that are in more advanced stages of the ACA program. Airports at these advanced stages implement more sophisticated and sustainable strategies. The current study encompasses airports at ACA levels 1 to 3+, whereas 66 airports worldwide have achieved ACA levels 4 and 4+ [65]. Additionally, the sensitivity of the topic affected the number of interviewees; some airports could only provide a limited number of respondents, which restricted the depth of insights obtained.

## 5 Conclusions

The transition management approach, utilising Loorbach's spheres tool [46], provides a valuable theoretical framework for guiding the aviation industry towards reducing air pollution with support to achieve the long-term sustainable goal of net zero emissions by 2050. Presently, the aviation sector lacks a definitive roadmap to reach this milestone, prompting ICAO to urge aviation stakeholders to take proactive measures nationally. Hence, aviation stakeholders are uncertain about the actions they can incorporate into their environmental policy. The novelty of this paper lies in its application of Loorbach's spheres tool in airport management, revealing how it logically supports the pursuit of the sustainability goal. This paper proposes a range of environmental strategies across the tactical, operational, and reflexive spheres, which airport stakeholders should incorporate into their environmental policies to realise this long-term milestone. By offering incremental progress and facilitating organisational changes, this paper assists international airports in navigating the complexities inherent in transitioning towards an uncertain, long-term goal.

The findings reveal that external factors such as bushfires, controlled burns, and road traffic influence air quality around airports [22]. Consequently, current airport practices of measuring air pollutant concentration may not accurately reflect their specific contribution. Thus, airports should assess air pollutant emissions from all airport sources and maintain an inventory for future decision-making. Furthermore, the findings indicate that airports prioritise a precise emission inventory with support from external consultants. However, this paper reveals the importance of initiating emission reduction strategies rather than solely refining inventory precision. Airports should measure the effectiveness of implemented strategies to motivate further reductions.

Airports currently rely on ICAO-suggested standard values for some airport sources, including Auxiliary Power Units (APUs), Ground Power Units (GPUs), and Ground Support Equipment (GSE) to estimate emissions due to data unavailability. A lack of data collaboration leads to inaccuracies as these ICAO values may not reflect each airport's specific conditions. Therefore, data collaboration among stakeholders, including air traffic controllers, airlines, and ground handling companies, should be implemented to enhance airfield efficiency and reduce environmental impacts.

Further research should model air pollutant emissions from aircraft, APUs, GPUs and GSE, to assess their influence on air quality. A methodology to estimate air pollutant emissions from aircraft ground idling delays should be developed to enhance the efficiency of airside ground operations, enabling practitioners to identify the emissions for which they are responsible. The environmental strategies outlined in this paper are 1) Collaborating data-sharing among stakeholders, including airport operators, ground handlers, airlines and air traffic controllers; 2) Evaluating emissions from aircraft ground idling delays; 3) Advancing in the Airport Carbon Accreditation program; 4) Assessing air pollutant emissions directly emitted from airport sources; 5) Maintaining an air pollutant emissions inventory. These strategies have the potential to strengthen environmental policies for airports and airlines, supporting the aviation industry to achieve net-zero emissions by 2050.

## CRediT authorship contribution statement

**Manori Dissanayaka:** Writing – review & editing, Writing – original draft, Visualization, Validation, Software, Resources, Project administration, Methodology, Investigation, Formal analysis, Data curation, Conceptualization. **Tim Ryley:** Writing – review & editing, Validation, Supervision, Resources, Project administration. **Bojana Spasojevic:** Writing – review & editing, Validation, Supervision, Resources, Project administration. **Savindi Caldera:** Writing – review & editing, Validation, Supervision, Resources, Project administration.

## Data availability statement

The raw data for this study consist of interview transcripts that may include confidential information. As participants did not provide explicit consent for public data sharing, we are unable to release the full transcripts. However, relevant de-identified excerpts have been included in this paper to support the findings.

## Declaration of generative AI and AI-assisted technologies in the writing process

During the preparation of this work, the lead author used ‘ChatGPT’ to improve language and readability. After using this service, the authors reviewed and edited the content as needed and take full responsibility for the publication's content.

## Funding

This research received no external funding.

## Declaration of competing interest

I confirm that there are no conflicts of interest, whether financial or non-financial, that could be perceived as affecting the integrity of the research or the objectivity of the conclusions presented in the manuscript. The authors declare no conflict of interest.

## Appendix 1 – Questionnaire

1. What is your involvement in sustainable aviation/aircraft operations? How long have you been involved in it?
2. What environmental strategies do you use to reduce emissions?
3. What are the expected outcomes of these environmental strategies?
4. How do you benchmark the performance of environmental strategies?
5. What are the challenges faced when implementing environmental strategies?
6. How do you calculate CO_2_ emissions from aircraft and other sources?
7. How do you measure the impacts on air quality from airport sources?
8. Which sources have the most influence on local air quality?
9. How does the airport record the operation time of aircraft according to the different flight stages? (Turnaround, pushback, taxiing out, take-off roll, take-off, climb, descent, landing, and taxing in)
10. How does the airport track the exact time of aircraft ground idling delays?
11. How does the airport record the operational time of APU, GPU, and GSE?
12. What are the airport/airline policies on APU and GPU operations?
13. What is your opinion about the reasons for ground idling delays?
14. What is your opinion about strategies used/would be used to reduce ground idling delays?
